# Contributions to the knowledge of subterranean trechine beetles in southern China’s karsts: five new genera (Insecta, Coleoptera, Carabidae, Trechinae)

**DOI:** 10.3897/zookeys.564.6819

**Published:** 2016-02-16

**Authors:** Mingyi Tian, Sunbin Huang, Xinhui Wang, Mingruo Tang

**Affiliations:** 1 Department of Entomology, College of Agriculture, South China Agricultural University, No. 483 Wushan Road, Guangzhou, 510642, China

**Keywords:** New genus, new species, new combination, cavernicolous, ground beetle, Guizhou, Yunnan, Guangxi, Hunan

## Abstract

Recent discoveries reveal that southern China’s karsts hold the most diverse and morphologically modified subterranean trechine beetles in the world, albeit the first troglobitic blind beetle was only reported in the early 1990’s. In total, 110 species belonging to 43 genera of cavernicolous trechines have hitherto been recorded from the karsts of southern China, including the following five new genera proposed below: *Shiqianaphaenops* Tian, **gen. n.**, to contain two species: *Shiqianaphaenops majusculus* (Uéno, 1999) (= *Shenaphaenops majusculus* Uéno, 1999, **comb. n.**), the type species from Cave Feng Dong, Shiqian, Guizhou, and *Shiqianaphaenops cursor* (Uéno, 1999) (= *Shenaphaenops cursor* Uéno, 1999, **comb. n.**), from Cave Shenxian Dong, Shiqian, Guizhou; and the monotypic *Dianotrechus* Tian, **gen. n.** (the type species: *Dianotrechus gueorguievi* Tian, **sp. n.**, from Cave Dashi Dong, Kunming, Yunnan), *Tianeotrechus* Tian & Tang, **gen. n.** (the type species: *Tianeotrechus trisetosus* Tian & Tang, **sp. n.**, from Cave Bahao Dong, Tian’e County, Guangxi), *Huoyanodytes* Tian & Huang, **gen. n.** (the type species: *Huoyanodytes tujiaphilus* Tian & Huang, **sp. n.**, from Longshan, Hunan) and *Wanhuaphaenops* Tian & Wang, **gen. n.** (the type species: *Wanhuaphaenops zhangi* Tian & Wang, **sp. n.**, from Cave Songjia Dong, Chenzhou, Hunan).

## Introduction

China is long known to support with the largest karst landscapes and ecosystems in the world (Waltham 2009). Like in many other fields of natural sciences, however, modern speleological activities in China started much later than in other countries of Europe, North America or Japan, although the well-known Ming Dynasty traveler Xu Xiake made important contributions to cave surveys already during the first half of the 17^th^ century. While in many countries caves are described and registered (e.g. more than 34,000 caves in Italy, see Stoch 2002), there is still no available cave database in China, where only rather few caves are well explored and surveyed. The situation is the same for cave biology. In China, cave-dwelling animals have been continuously reported since the 1980’s, mostly by foreign researchers, but there is still a long way to go. With the exception of several vertebrates such as fishes, bats and frogs, almost nothing is known about troglobitic invertebrates presented in the Red Data book of China or on the list of endangered species which are strictly protected by law in China (Wang and Xie 2005).

However, this situation is changing. Discoveries during last two decades reveal that the karsts of southern China host the globe’s most diverse fauna of subterranean trechine beetles, including that at the generic level. Up to date, 38 genera and 106 species of cave trechines have been recorded from southern China. Below is a checklist of cavernicolous trechine genera known from southern China’s karsts, coupled with data on their species diversity and geographical ranges.

*Agonotrechus* Jeannel, 1923: only a single troglophilous species recorded from a cave in western Hubei (Deuve 1999);

*Aspidaphaenops* Uéno, 2006: three species, all troglobitic and all known from Qianxinan, Guizhou (Uéno 2006b);

*Bathytrechus* Uéno, 2005: one species, troglobitic, in Leye, northwestern Guangxi (Uéno 2005a);

*Boreaphaenops* Uéno, 2002: a single species, troglobitic, in Shennongjia, western Hubei (Uéno 2002, 2010);

*Cathaiaphaenops* Deuve, 1996: six species, troglobitic, known from Hunan, Hubei and Chongqing (Deuve 1996, 1999, Uéno 2000b);

*Cimmeritodes* Deuve, 1996: two species, troglobitic, one in Longshan, northwestern Hunan, the other in Quzhou, eastern Zhejiang (Deuve 1996, Uéno 2006b, Deuve and Tian 2015);

*Dongodytes* Deuve, 1993: 12 species, highly modified, troglobitic, in several counties of northwestern Guangxi (Deuve 1993, Uéno 1998, 2005c, Tian 2011, Tian et al. 2014);

*Giraffaphaenops* Deuve, 2002: two species, extremely modified, troglobitic, in several caves in Leye and a cave in Tianlin, northwestern Guangxi respectively (Deuve 2002, Uéno 2003b, Tian and Luo 2015);

*Guiaphaenops* Deuve, 2002: one species, troglobitic, in Lingyun, northwestern Guangxi (Deuve 2002; Uéno 2006a);

*Guizhaphaenops* Vigna Taglianti, 1997: 11 species in two subgenera, troglobitic, in Guizhou and Yunnan (Vigna Taglianti 1997, Deuve 1999, 2001, Uéno 1998, 2000a, Uéno and Ran 2004, Deuve and Quéinnec 2014);

*Jiangxiaphaenops* Uéno & Clarke, 2007: a single species, troglobitic, known from Wannian, Jiangxi (Uéno and Clarke 2007);

*Jiulongotrechus* Tian, Huang & Wang, 2015: a single species, troglobitic, known from Tongren, eastern Guizhou (Tian et al. 2015);

*Junaphaenops* Uéno, 1997: a unique species, troglobitic, known from Kunming, the capital City of Yunnan Province (Uéno 1997);

*Libotrechus* Uéno, 1998: two species, troglobitic, in southernmost Guizhou and northern Guangxi (Uéno 1998, Lin and Tian 2014);

*Luoxiaotrechus* Tian & Yin, 2013: two species, troglobitic, from eastern Hunan (Tian and Yin 2013, Tian and Huang 2015);

*Microblemus* Uéno, 2007: a single species, troglobitic, in Jinhua, eastern Zhejiang (Uéno 2007);

*Minimaphaenops* Deuve, 1999: a single species, troglobitic, in Fengjie, Chongqing (Deuve 1999);

*Oodinotrechus* Uéno, 1998: Three species, troglobitic, two in southernmost Guizhou and northernmost Guangxi (Uéno 1998, Tian 2014), one in Pingle, northeastern Guangxi (Sun and Tian 2015);

*Pilosaphaenops* Deuve & Tian, 2008: 2-3 species, highly modified, troglobitic, in southernmost Guizhou and northernmost Guangxi (Uéno 2002, Deuve and Tian 2008, Tian 2010);

*Plesioaphaenops* Deuve & Tian, 2011: a single species, troglobitic, in Longlin, western Guangxi (Deuve and Tian 2011);

*Qianaphaenops* Uéno, 2000: six species, troglobitic, from northeastern Guizhou (Uéno 2000c, Tian and Clarke 2012, Tian et al. 2015);

*Qianotrechus* Uéno, 2000: five species, troglobitic, in northern Guizhou and southeastern Sichuan (Uéno 2000c, 2003a);

*Satotrechus* Uéno, 2006: two troglobitic species, one in southwestern Guizhou, the other in northwestern Guangxi (Uéno 2006b, Deuve and Tian 2011);

*Shenaphaenops* Uéno, 1999: one species, troglobitic, in northwestern Guizhou (Uéno 1999a). The other two species originally assigned to this genus (Uéno 1999b) are transferred into a new genus described below;

*Shuaphaenops* Uéno, 1999: one species, troglobitic, in southern Chongqing (Uéno 1999c);

*Shilinotrechus* Uéno, 2003: two species, troglobitic, from eastern Yunnan (Uéno 2003a, Huang and Tian 2015);

*Sichuanotrechus* Deuve, 2005: five species, troglobitic, in northern Sichuan (Deuve 2005, Uéno 2006a, 2008, Huang and Tian 2015);

*Sidublemus* Tian & Yin, 2013: one species, troglobitic, from southeastern Hunan (Tian and Yin 2013);

*Sinaphaenops* Uéno & Wang, 1991: nine species in three subgenera, highly modified, troglobitic, from western to southern Guizhou, and northernmost Guangxi (Uéno and Wang 1991, Magrini et al. 1997, Uéno and Ran 1998, Uéno 2002, Deuve and Tian 2014, Tian and Huang 2015);

*Sinotroglodytes* Deuve, 1996: two species, troglobitic, in northwestern most Hunan (Deuve 1996, Uéno 2009);

*Superbotrechus* Deuve & Tian, 2009: a single species, troglobitic, in western Hubei (Deuve and Tian 2009);

*Toshiaphaenops* Uéno, 1999: two species, troglobitic, from northwestern Hunan and western Hubei (Uéno 1999a);

*Trechiotes* Jeannel, 1954: three species, troglophilous, in Guizhou and Guangxi (Deuve et al. 1999, Deuve 1995, Uéno 2007);

*Uenotrechus* Deuve & Tian, 1999: one species, highly modified, troglobitic, in southern Guizhou and northern Guangxi (Deuve et al. 1999, Deuve and Tian 2010);

*Wulongoblemus* Uéno, 2007: one species, troglobitic, in western Zhejiang (Uéno 2007);

*Yanzaphaenops* Uéno, 2010: a single species, highly modified, troglobitic, in Shennongjia, western Hubei (Uéno 2005b, 2010);

*Yunotrechus* Tian & Huang, 2014: a single troglobitic species from southernmost Yunnan (Tian and Huang 2014);

*Zhijinaphaenops* Uéno & Ran, 2002: five species, troglobitic, in western and central Guizhou (Uéno and Ran 2002; Deuve and Tian 2015).

Uéno (1999a) set up the genus *Shenaphaenops* based on a single female collected from a limestone cave called Shen Dong (actually, the complete name of the cave is Shendong Migong) in Shuicheng County, northwestern Guizhou Province. Later, he found two new species from caves in Shiqian County, northeastern Guizhou and described them in *Shenaphaenops*, even though he realized that both two counties lay very far from each other, and the beetles found in Shiqian showed some peculiar features, in particular, a dilated protarsomere 1 in both sexes, a character never seen in other trechines (Uéno 1999b). We visited the type localities in both counties twice in 2014 and 2015, respectively, and successfully collected a male of *Shenaphaenops humeralis* Uéno, 1999, the type species, as well as an abundant material of *Shenaphaenops majusculus* Uéno, 1999. The newly collected samples provided enough evidence to show that both of these species are sufficiently different in many characters of generic importance to warrant the placement of the two known species from Shiqian into a new genus, not *Shenaphaenops*.

Thanks to Dr. Borislav V. Gueorguiev (National Museum of Natural History, Sofia, Bulgaria), we received a very peculiar trechine for study and as a gift. This female specimen was collected in a limestone cave called Dashi Dong in a suburb of Kunming, the capital city of Yunnan Province in autumn 2011 during a China-Bulgaria joint cave exploration in Yunnan organized by Prof. Fan Zhang (Yunnan Institute of Geography, Yunnan University). This interesting anophthalmic beetle appears to be new both at the specific and generic levels.

Among the new findings of cave-dwelling trechines during our cave surveys in Guangxi in July and August 2015, perhaps the most interesting was an unexpected species collected in Cave Bahao Dong, Tian’e County. It is sympatric with *Dongodytes giraffa* Uéno, 2005, an extremely troglomorphic beetle, but shows very unusual morphological characters not seen in any other cavernicolous trechines known in China, such as the right mandible being quadridentate, the pronotal lateral borders invisible from above and the elytra with three dorsal pores.

In July 2014, a single female trechine was discovered in the famous Huoyan Karst, Longshan County, northwesternmost Hunan Province. It lives there together with *Cathaiaphaenops*, *Sinotroglodytes*, *Cimmeritodes* and *Toshiaphaenops* species. However, this semi-aphaenopsian beetle is very different from the other sympatric Trechini, with several peculiar characters such as a very long and tube-like head, strongly dilated femora, very convex pronotum and elytra, and a strange elytral chaetotaxy, in which the humeral group of marginal umbilicate series is composed of five, not four, pores.

In late August 2015, we were invited to undertake a cave biodiversity survey in the Wanhuayan cave system, southern Hunan, as part of the cave exploration activities headed by Prof. Yuanhai Zhang (Institute of Karst Geology, Chinese Academy of Geological Sciences, Guilin). Several specimens of a blind trechine species were collected. This peculiar troglobitic species is probably related to *Shenaphaenops* or even the *Sinaphaenops* complex, but with many different characteristics of generic importance.

In order to properly assess the above trechine species, five new genera are established below, including the proposal of two new combinations for both Shiqian species formerly treated in *Shenaphaenops*, and the description of four new species forming four monotypic genera from limestone caves in Yunnan, Guangxi and Hunan, respectively.

## Material and methods

The beetle specimens were collected by using an aspirator inside the cave, and kept in 55% ethanol before study. Dissections and observations were made under a Leica S8AP0 microscope. Dissected genital pieces, including the median lobe and parameres of the aedeagus, were glued onto small transparent plastic plates and pinned under the specimen they belonged to. Habitus pictures were taken by means of a Keyence VHX-5000 digital microscope. Genital pictures were taken using a Canon EOS 40D camera connected to a Zeiss AX10 microscope, and then stacked and processed by means of Adobe Photoshop CS5 software. Distribution maps created using Mapinfo software.

The length of the body was measured from the apex of the right mandible (in open position) to the elytral apex; the width of the body was taken as the maximum width of the elytra.

Abbreviations of other measurements used in the text are as follows:

HLm
 length of head including mandibles, from apex of right mandible to occipital suture 


HLl
 length of head excluding mandibles, from front of labrum to occipital suture 


HW
 maximum width of head 


PrL
 length of prothorax, along the median line 


PnL
 length of pronotum, as above 


PrW
 maximum width of prothorax 


PnW
 maximum width of pronotum 


PfW
 width of pronotum at front 


PbW
 width of pronotum at base 


EL
 length of elytra, from base of scutellum to elytral apex 


EW
 maximum width of combined elytra 


## Taxonomy

### Shiqianaphaenops

Taxon classificationAnimaliaColeopteraCarabidae

Tian gen. n.

http://zoobank.org/D7A2BFB8-59A2-4BA4-A26C-630F9E5B7488

#### Type species.

*Shenaphaenops majusculus* Uéno, 1999 (Cave Feng Dong, Shiqian, Guizhou).

#### Diagnosis.

Medium-sized aphaenopsian trechine beetles, with sparsely pubescent body, elongated head, reduced frontal furrows, tridentate right mandible, 4-setose mentum, tumid propleura, widened 1^st^ protarsomere and bisetose on each of abdominal ventrites.

#### Generic characteristics.

Medium-sized aphaenopsian trechines, yet not too highly modified morphologically; eyeless, unpigmented and apterous; body slender and elongate, with slender and long appendages; covered with sparse pubescence, hairs being much longer on head and pronotum than on elytra; head elongate, longer than prothorax, much longer than wide; frontal furrows short, with two pairs of supra-orbital pores; right mandible tridentate; labial suture clear; mentum 4-setose, tooth simple and short, blunt at apex; submentum 9-setose; antennae long, nearly extending to elytral apex; prothorax longer than wide, propleura visible from above; pronotum elongate, longer than wide, widest near front; only anterior pairs of lateromarginal setae; elytra elongate-ovate, strongly convex, shoulders distinct and rounded, lateral margins ciliated throughout; striae faintly impressed; two dorsal and the pre-apical pores present on each elytron; humeral group of marginal umbilicate pores not aggregated; protibia without longitudinal groove externally; 1^st^ protarsomere in both sexes widened and angularly produced externally, with a row of comb-like setae on ventral side; 1^st^ and 2^nd^ protarsomeres modified in male; ventrite VII with two pairs of setae in both sexes.

#### Remarks.

**Figure 1.**
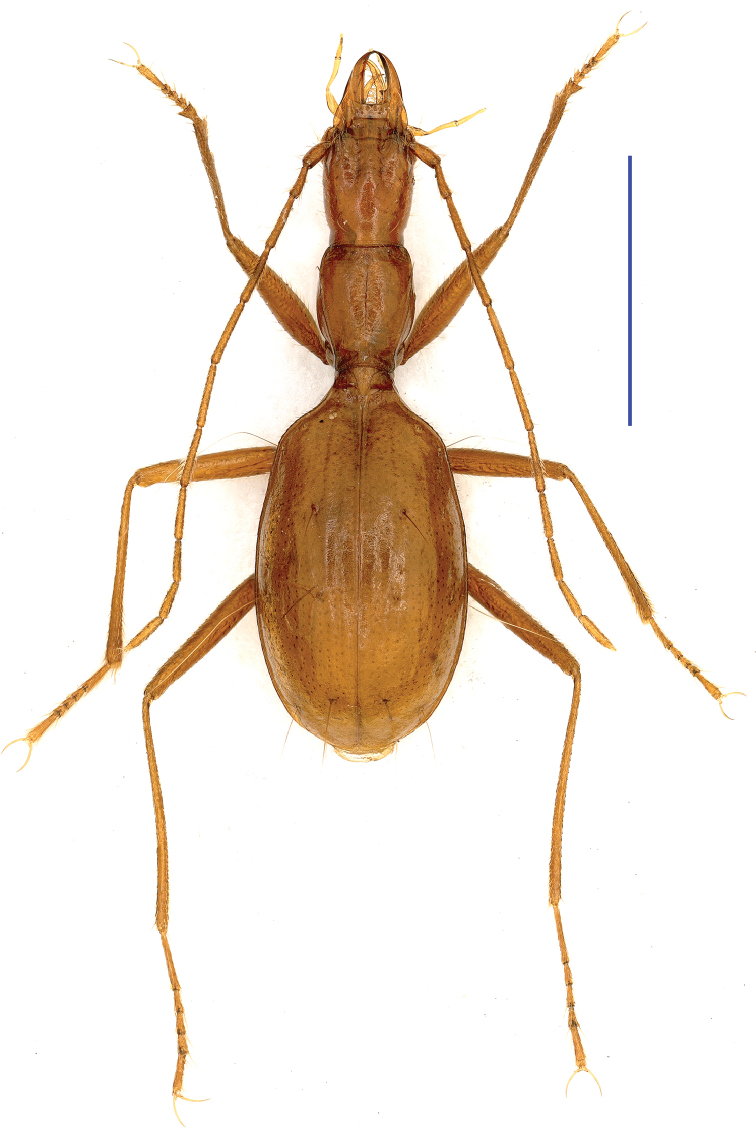
Habitus of *Shiqianaphaenops majusculus* (Uéno, 1999), comb. n., male Scale bar: 2.0 mm.

**Figure 2.**
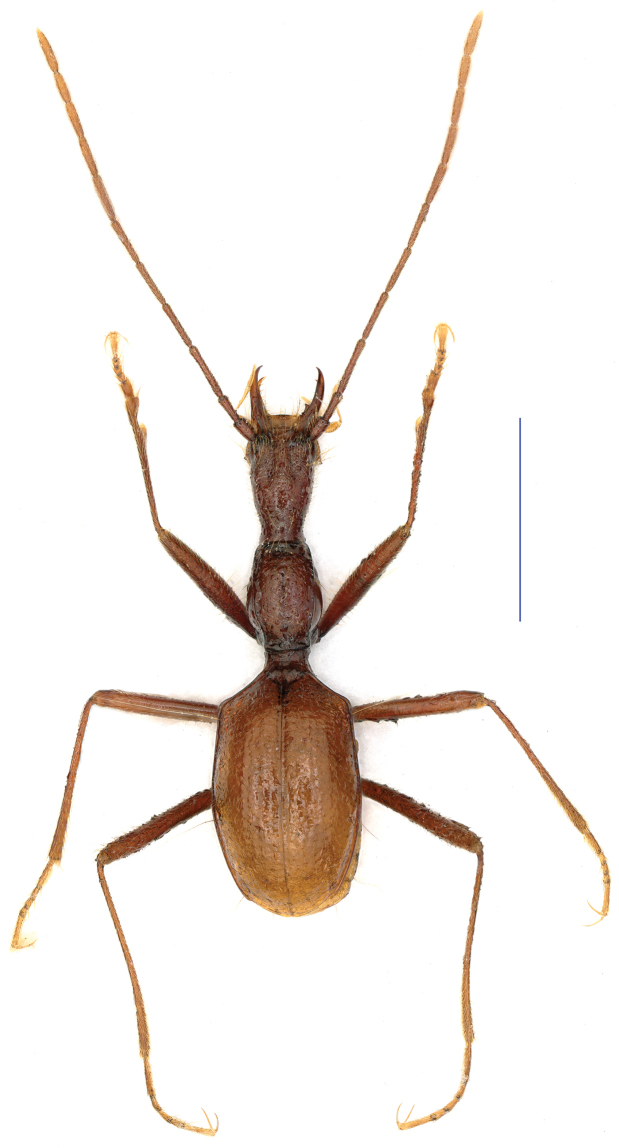
Habitus of *Shenaphaenops humeralis* Uéno, 1999, male Scale bar: 2.0 mm.

**Figure 3.**
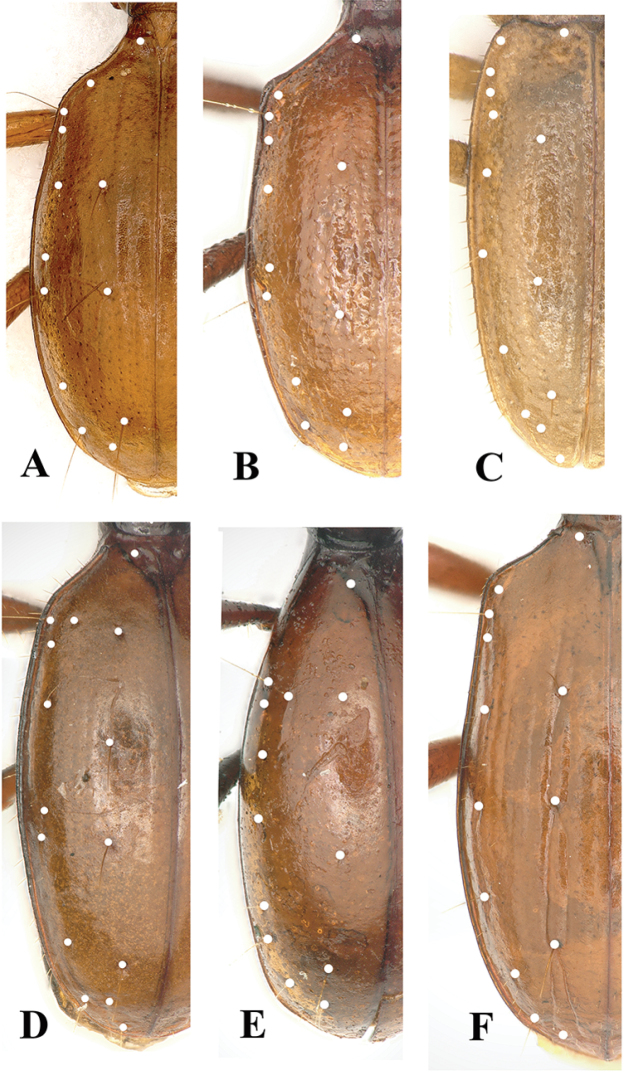
Elytral chaetotaxy **A** *Shiqianaphaenops majusculus* (Uéno, 1999), comb. n. **B** *Shenaphaenops humeralis* Uéno, 1999 **C** *Dianotrechus gueorguievi* Tian, gen. n., sp. n. **D** *Tianeotrechus trisetosus* Tian & Tang, gen. n., sp. n. **E** *Huoyanodytes tujiaphilus* Tian & Huang, gen. n., n. sp. **F** *Huoyanodytes tujiaphilus* Tian & Huang, gen. n., sp. n. **G** *Wanhuaphaenops zhangi* Tian & Wang, sp. n.

**Figure 4.**
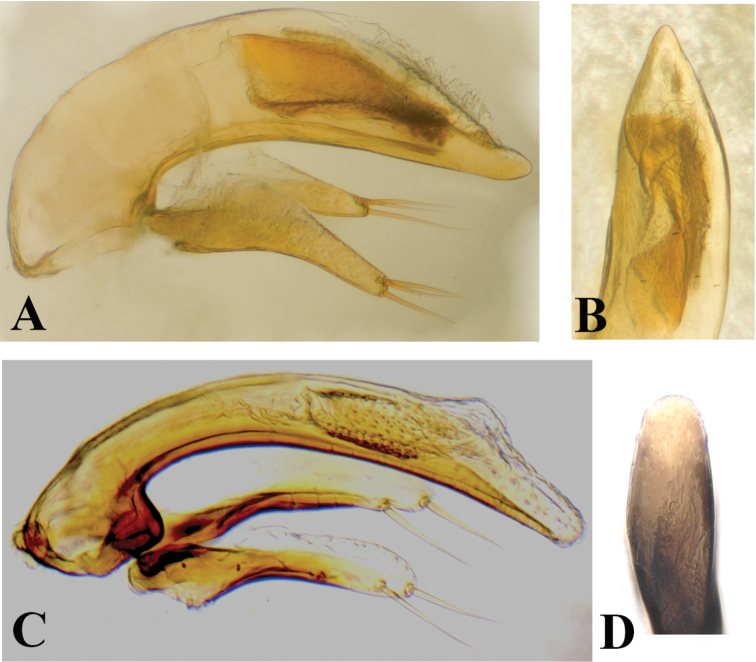
Male genitalia **A** and **C** median lobe, lateral view **B** and **D** apical lobe, dorsal view **A** and **B** *Shiqianaphaenops majusculus* (Uéno, 1999), comb. n. **C** and **D** *Shenaphaenops humeralis* Uéno, 1999.

Uéno (1999) hesitantly treated the two trechine species found in Shiqian County, eastern Guizhou as members of the genus *Shenaphaenops* Uéno, 1999, which had been set up based on a single female collected in Shuicheng County, northwestern Guizhou. However, he realized some striking differences and the geographical gap between the Shiqian species and the type species *Shenaphaenops humeralis* Uéno, 1999. Apart from the somewhat similar general body configurations in both *Shiqianaphaenops* gen. n. (Fig. 1) and *Shenaphaenops* (Fig. 2), many character states of generic importance are different, such as: (1) 1^st^ protarsomere in both sexes widened apically and distinctly produced externally, covered with a ctenidium structure ventrally in *Shiqianaphaenops* which, amongst trechines, is only found in this genus; (2) right mandible tridentate in *Shiqianaphaenops*, versus bidentate in *Shenaphaenops*; (3) labial suture clear in *Shiqianaphaenops*, but completely missing in *Shenaphaenops*; (4) ventrite VII in male bisetose in *Shiqianaphaenops*, versus 4-setose in *Shenaphaenops*; (5) in male, both 1^st^ and 2^nd^ protarsomeres modified in *Shiqianaphaenops*, versus only 1^st^ protarsomere being modified in *Shenaphaenops*; (6) pubescence weaker and sparser, hairs much longer on head and pronotum than on elytra in *Shiqianaphaenops*, versus generally denser and hairs being of same length in *Shenaphaenops*; (7) base of pronotum much narrower than front in *Shiqianaphaenops*, versus only slightly narrower in *Shenaphaenops*; (8) elytra broader, with humeral angles rounded in *Shiqianaphaenops* (Fig. 3A), versus more slender, with humeral angles distinctly angular in *Shenaphaenops* (Fig. 3B); (9) marginal sides of elytra ciliate throughout, versus smooth in *Shenaphaenops*; (10) in *Shiqianaphaenops*, median lobe of aedeagus stout and thick, broadly rounded dorsally, basal part comparatively small, with an indistinct sagittal aileron, inner sac armed with a very large copulatory piece; apical lobe contracted at apex, roundly blunt in lateral view, but pointed in dorsal view; parameres thin, much shorter than median lobe, each bearing three long setae at apex (Fig. 4A, B). In contrast, median lobe of aedeagus in *Shenaphaenops* slender and thin, gently expanded dorsally, basal part comparatively large, with a small but distinct sagittal aileron; apical lobe indistinctly contracted at apex, obtuse in lateral view, broad in dorsal view; inner sac armed with a large copulatory piece; parameres shorter than median lobe, left one slightly longer than right one, each bearing two long setae at apex (Fig. 4C, D).

#### Etymology.

Shiqian+Aphaenops, to indicate that both known members of this genus occur in Shiqian County, eastern Guizhou Province. Gender masculine.

#### Range.

**Figure 5.**
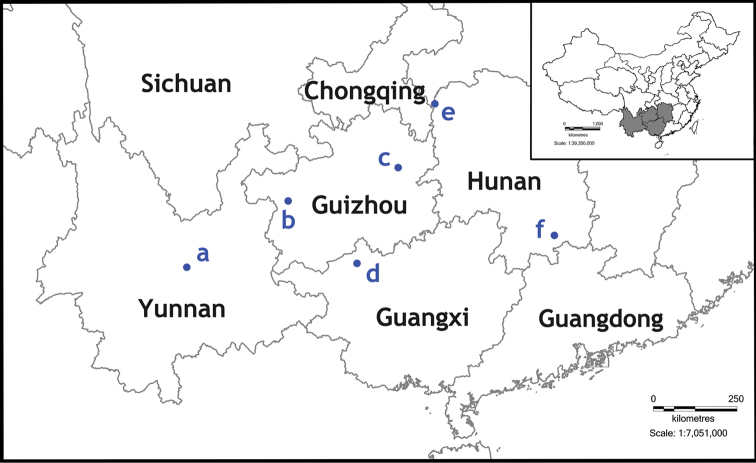
Distribution of the trechine beetles **a** *Dianotrechus gueorguievi* Tian, gen. n., sp. n. **b** *Shenaphaenops humeralis* Uéno, 1999 **c** *Shiqianaphaenops majusculus* (Uéno, 1999), comb. n. **d** *Tianeotrechus trisetosus* Tian & Tang, gen. n., sp. n. **e** *Huoyanodytes tujiaphilus* Tian & Huang, sp. n. **f** *Wanhuaphaenops zhangi* Tian & Wang, gen. n., sp. n.

China (eastern Guizhou) (Fig. 5c).

**Figure 6.**
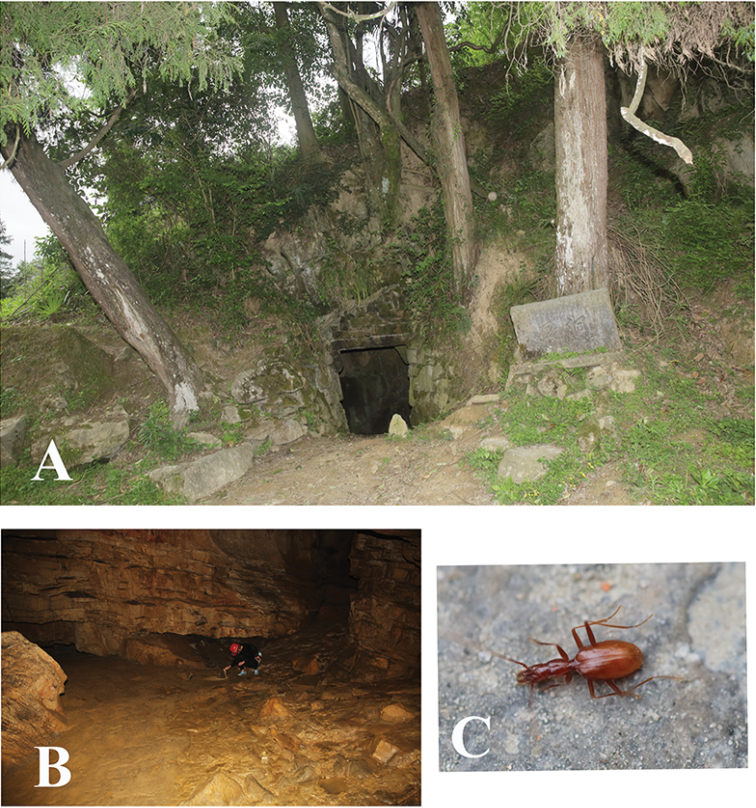
Cave Feng Dong, type locality of *Shiqianaphaenops majusculus* (Uéno, 1999), comb. n. **A** entrance **B** cave chamber, to show where the beetles were collected **C** a wandering individual in cave.

Known so far by two very similar species from two caves in Shiqian County: Shenxian Dong and Feng Dong. Since Shenxian Dong lies very close to Feng Dong, only about one kilometre in distance across a shallow valley, with still another cave just between them, all three caves may prove belong to a same cave system. Perhaps this is why both Uéno’s species from Shiqian County are not too different from each other. Feng Dong is a large and beautiful cave (Fig. 6A, B). The beetle (Fig. 6C) is sympatric with millipedes, crickets and frogs.

#### Material examined.

*Shenaphaenops humeralis* Uéno, 1999: a male, Cave Shendong Migong (Shendong in the original description), Muqiao, Laoyingshan, Shuicheng County, northwestern Guizhou, 26°35'15"N/ 104°59'47"E, 1910 m in altitude, VIII-22-2014, Mingyi Tian leg., deposited in the insect collections of South China Agricultural University (SCAU).

*Shiqianaphaenops majusculus* (Uéno, 1999), comb. n.: 3 males, V-1-2015, Cave Feng Dong, Tangshan, Shiqian County, 27°29'10"N/108°15'23"E, 700 m in altitude, Mingyi Tian & Jingli Cheng leg., in SCAU; 6 males & 4 females, ibid., VIII-1-2015, same collectors, in SCAU.

*Shiqianaphaenops cursor* (Uéno, 1999), comb. n.: We have not seen any material. We visited Cave Shenxian Dong, the type locality, in August 2015, but failed to catch anything. The cave is badly disturbed by human activities.

### Dianotrechus

Taxon classificationAnimaliaColeopteraCarabidae

Tian gen. n.

http://zoobank.org/844915FD-7B9B-421B-8143-0ACA207CEF54

#### Type species.

*Dianotrechus gueorguievi* Tian, sp. n. (Cave Dashi Dong, Kunming, Yunnan).

#### Diagnosis.

Small-sized and anophthalmic trechine beetles, with robust head, complete frontal furrows, bidentate right mandible, 4-setose mentum, fused mentum and submentum, short appendages, quadrate pronotum, widely spaced middle pores of the marginal umbilicate setiferious series and 6-setose ventrite VII in female.

#### Generic characteristics.

Small-sized, anophthalmic trechine; unpigmented and apterous; head robust, genae strongly convex laterally; frontal furrows entire, two pairs of supra-orbital pores; right mandible bidentate; mentum and submentum completely fused; submentum 7-setose, a shorter seta present in the middle; mentum 4-setose; antennae short and stout, extending to basal third of elytra; prothorax with propleura invisible from above; pronotum quadrate, two pairs of laterodorsal setae present; elytra elongate-ovate, moderately convex, without prehumeral angles, lateral margins gently expanded, ciliated throughout; punctate-striate, two dorsal and the pre-apical pores present, apical stria present, humeral group of marginal umbilicate pores not aggregated, 5^th^ and 6^th^ pores of middle group widely separated, 5^th^ pore strikingly shifted forward, much closer to 4^th^ than to 6^th^; scutellum small; legs short and stout; protibia without longitudinal groove externally; ventrite VII in female with three pairs of setae.

#### Remarks.

The most striking peculiarities of this new genus lie in the conformation of the pronotum and the chaetotaxal pattern on the elytra, especially the middle group of umbilicate pores, in which the 5^th^ pore is much closer to the 4^th^ than to the 6^th^. *Dianotrechus* gen. n. seems to be particularly close to *Shilinotrechus* Uéno, 2003, an anophthalmic trechine genus also recorded from eastern Yunnan, but the former genus differs from the latter by the following character states: small-sized (versus medium-sized in *Shilinotrechus*); right mandible bidentate (versus tridentate in *Shilinotrechus*); body shape nearly parallel-sided (versus fusiform in *Shilinotrechus*); head of anophthalmic type (versus aphaenopsian type in *Shilinotrechus*); ventrite VII with three pairs of setae in female (versus two pairs in *Shilinotrechus*). Some more differences are also evident in the conformation of the pronotum and elytra, as well as the chaetotaxy pattern of the marginal umbilicate series.

Compared to *Cimmeritodes* Deuve, 1996, a small-sized trechine genus originally reported from the Huoyan Karst of Longshan County, northwestern Hunan Province, but also occurring in Zhejiang (Deuve and Tian 2015), *Dianotrechus* gen. n. is easily distinguished by the bidentate right mandible (versus tridentate in *Cimmeritodes*), the quadrate pronotum (versus cordate in *Cimmeritodes*), the chaetotaxy pattern of the marginal umbilicate series, in which the 5^th^ is more distant from the 6^th^ than from the 4^th^ (reverse in *Cimmeritodes*), and ventrite VII has three pairs of setae in the female (two pairs in *Cimmeritodes*).

#### Etymology.

Dian + Trechus, “Dian” is a short name for Yunnan Province in Chinese. The name of the new genus reflects the occurrence of this cavernicolous trechine in Yunnan. Gender masculine.

#### Range.

China (eastern Yunnan) (Fig. 5a).

### Dianotrechus gueorguievi

Taxon classificationAnimaliaColeopteraCarabidae

Tian sp. n.

http://zoobank.org/1618C05C-2922-4288-94C0-AF26A23054B2

#### Holotype.

Female, labeled “China, Yunnan Province, Ermu Vill., Kunming District, Dashi Dong (Big Rock Cave), 24°49'13"N/102°27'56"E, 1940 m in altitude, XI-8-2011, B. Petrov leg.”, in SCAU.

#### Diagnosis.

A small, stout, yellowish brown beetle which is densely pubescent, with short fore body and appendages, convex head, not tumid propleura which invisible from above, rather flat elytra and coarsely punctate elytral striae.

#### Description.

**Figure 7.**
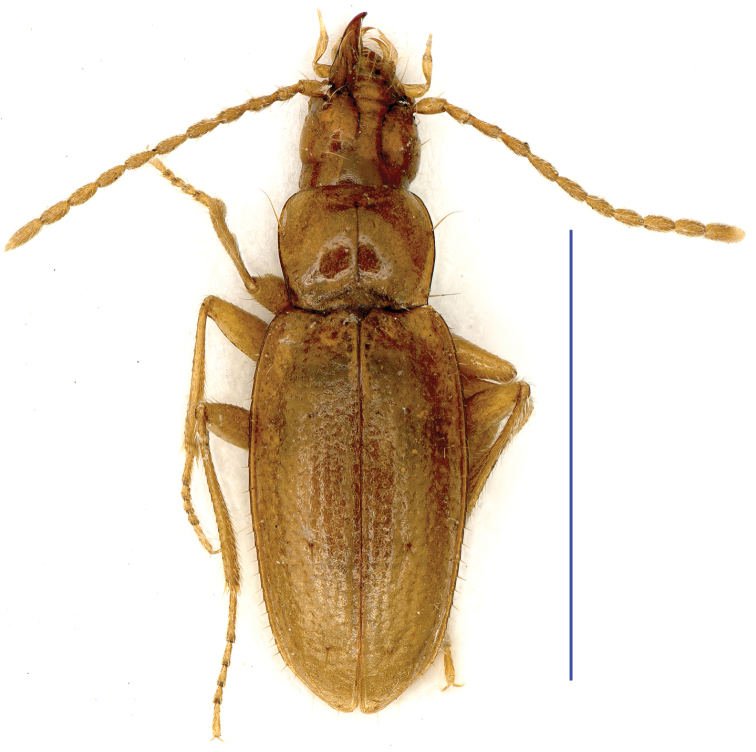
Habitus of *Dianotrechus gueorguievi* Tian, gen. n., sp. n., holotype, female Scale bar: 2.0 mm.

Length: 3.1 mm (including mandibles), width: 0.9 mm. Habitus as in Fig. 7

Whole body yellowish brown, with palps pale; head and pronotum shiny, elytra dim; frons and vertex glabrous, genae with several short hairs, pronotum with a few fine setae, whole elytra covered with erect setae, these being as long as those on genae; underside generally glabrous, smooth and polished, but a few short hairs present on ventrites II and IV, and in lateral areas of prosternum; microsculptural meshes vanishing on head and pronotum, densely and moderately engraved on elytra. Fore part of body much shorter than elytra, EL/(HLm+PnL) = 1.39.

Head short and stout, much longer than wide (including mandibles), HLm/HW = 1.44, or as long as wide (excluding mandibles), genae broadly convex, frons and vertex moderately convex, frontal furrows entire, strongly divergent backwards; both supra-orbital pores closely located, posterior ones almost on frontal furrows, distance between anterior and posterior pores shorter than that between supra-orbital furrows at the closest point, neck short and broad; clypeus 4-setose, labrum transverse, nearly straight at frontal margin, 6-setose; mandibles rather short; labial suture missing; mentum 4-setose (two laterally and two at base of mental tooth); mentum tooth simple, very short, broad at apex; basal emargination wide and rather deep; ligula small and short, adnated to paraglossae, widened at apex, 6-setose; palps stout and short, penultimate joints much stouter than apical ones; 3^rd^ maxillary palpomere slightly longer than 4^th^, labial palpomere 2^nd^ distinctly longer than 3^rd^, bisetose at inner margin, with two additional setae in outer apical parts; 3^rd^ maxillary palpomere with two tiny setae near apex; suborbital pores present, located in median portion of ventral genae, lying far from base of head; antennae short and stout, wholly pubescent, 1^st^ antennomere stouter than others, 1^st^, 2^nd^ and 4^th^-10^th^ subequal in length, 3^rd^ slightly longer than 2^nd^, but slightly shorter than 11^th^.

Prothorax: propleura not tumid, invisible from above; pronotum transverse, PnL/PnW = 0.84, wider than head, PnW/HW = 1.27, much shorter than head (including mandibles), HLm/PnL = 1.44, or as long as head (excluding mandibles); disc moderately convex; widest at about middle where lateral sides slightly expanded but remaining nearly parallel-sided, reflexed near hind angles; fore lateromarginal seta located at a little before middle, basal one a little before hind angle; fore angles rounded, basal ones rectangular and pointed; base as wide as front; front almost straight, base nearly straight medially, obtusely sinuate near hind angles; median line fine and well-defined, reaching front margin, but ending before basal transverse impression, the latter being distinctly marked and connected to basal foveae; front transverse impression unclear. Scutellum small and short.

Elytra elongate, slender, moderately convex, wider than pronotum, almost twice as long as wide, EL/EW =1.93, widest at about middle of elytra, gently narrowed towards base and subapex; base wide, shoulders rounded, prehumeral angles missing; apex of each elytron rounded; disc moderately convex, striae coarsely punctate, intervals slightly convex; 1^st^-4^th^ striae and apical striae well-marked, 1^st^-3^rd^ striae complete, 4^th^ finished at level before median dorsal pore; other striae wanting; basal pore present, lying near basal margin and on side of scutellum; both dorsal pores located on 4^th^ intervals, at about basal third and a little behind the middle of elytra, respectively, pre-apical pore located at apical fusion of 2^nd^ and 3^rd^ striae, level to ending point of apical stria, about twice as far from apex as from suture; marginal umbilicate series with 1^st^, 2^nd^, 6^th^ and apical pores close to marginal gutter, 2^nd^-4^th^ pores equidistant, but 1^st^ more isolated; 5^th^ pore widely removed away from 6^th^ and closer to 4^th^ pore.

Legs moderately long, covered with dense and short hairs; protarsi short, 1^st^ tarsomere not distinctly wider than others, longer than 2^nd^ and 3^rd^ combined, but shorter than 2^nd^-4^th^ combined; meso- and metatarsi longer, 1^st^ tarsomere as long as 2^nd^-4^th^ combined, respectively. Ventrites IV-VI each with a pair of paramedian setae, ventrite VII in female with three pairs of setae.

Male: Unknown.

#### Etymology.

In honour of Dr. Borislav V. Guéorguiev (National Museum of Natural History, Sofia, Bulgaria), an expert in Carabidae.

#### Distribution.

**Figure 8.**
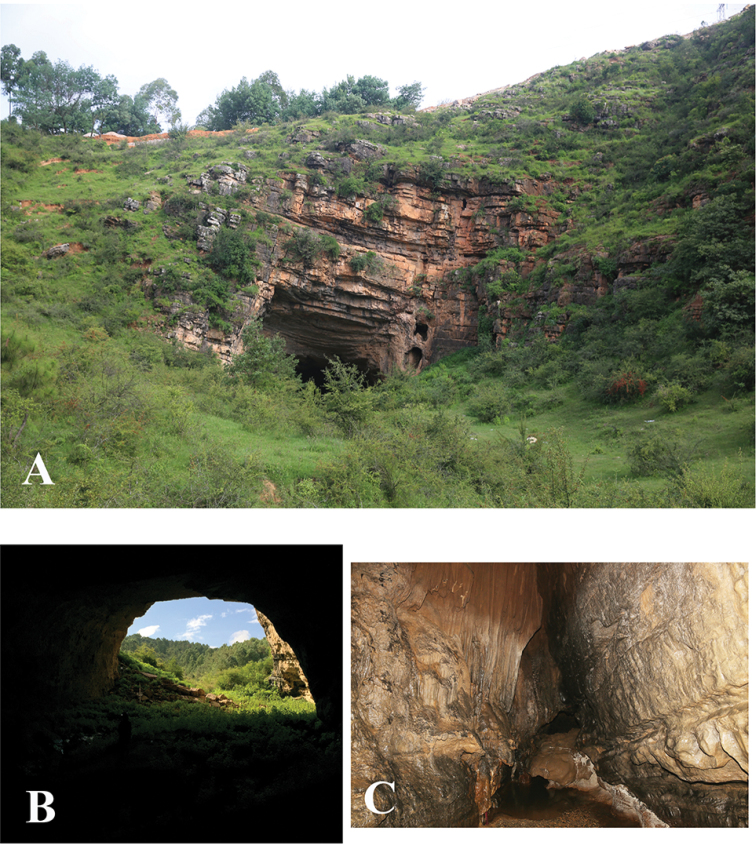
Cave Dashi Dong, type locality of *Dianotrechus gueorguievi* Tian, gen. n., sp. n. **A** entrance, outside view **B** entrance, inside view **C** main passage.

China (Yunnan) (Fig. 5a). Known only from the limestone Cave Dashi Dong in a western suburb of Kunming City (Fig. 8A–C).

Dashi Dong is located more than 1 km away from Ermu Village, Xianjie Zhen, Anning, Kunming. The opening of this cave is 27 m wide and 17 m high. Its total length is 1394 m and the total depth is 39.30 m. The temperature in the dark parts is 21 °C. The unique beetle was collected in the dark area. In order to find more specimens of this interesting beetle, we visited this cave three times in July 2014, July 2015 and August 2015, but of no avail.

### Tianeotrechus

Taxon classificationAnimaliaColeopteraCarabidae

Tian & Tang gen. n.

http://zoobank.org/49A4C222-27E7-4A48-8A0D-42C391FF432C

#### Type species.

*Tianeotrechus trisetosus* Tian & Tang, sp. n. (Bahao Dong, Tian’e County, Guangxi).

#### Diagnosis.

Medium-sized cave beetles, with typical aphaenopsian head, reduced frontal furrows, quadridentate right mandible, evident labial suture, bisetose mentum, robust pronotum, invisible propleura from above though which is tumid, and strongly covex elytra which have three pairs of dorsal setiferious pores.

#### Generic characteristics.

**Figure 9.**
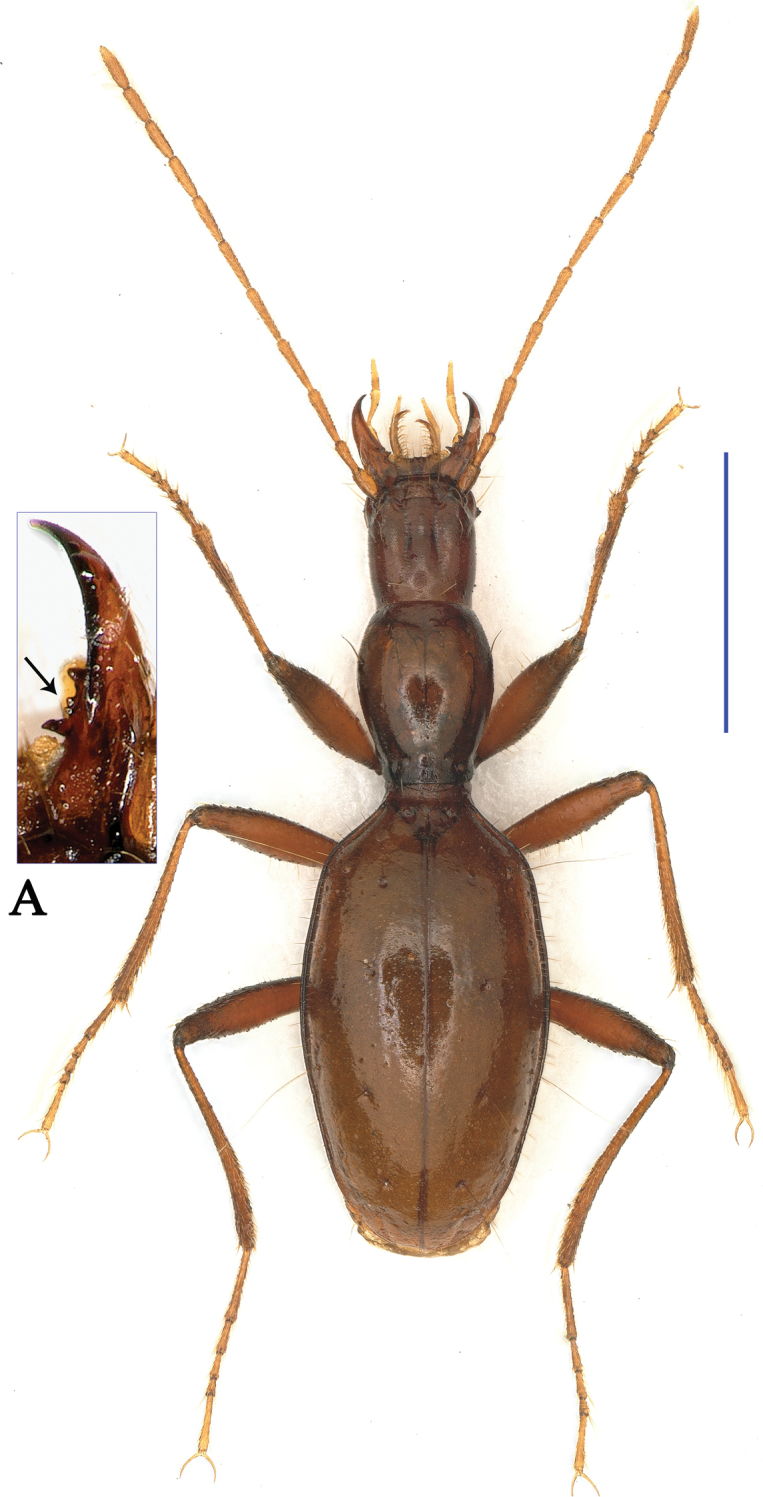
Habitus of *Tianeotrechus trisetosus* Tian & Tang, gen. n., sp. n., holotype, male Scale bar: 2.0 mm, **A** enlarged right mandible to show the quadrisetose teeth

Medium-sized and semi-aphaenopsian trechines, eyeless, unpigmented and apterous; head evidently aphaenopsian, with incomplete frontal furrows and a somewhat elongated head, with two pairs of supra-orbital pores; mandibles developed, right mandible quadridentate, molar and retinacular teeth more developed than premolar tooth which is bifid (Fig. 9A); mentum and submentum well separated by labial suture; mentum bisetose, distinctly concave, mental tooth short and thick, bifid apically; submentum provided with a row of seven setae, median one minute and much shorter than others; antennae fairly short, reaching a little beyond middle of elytra; pronotum robust, longer than wide, sides expanded at apical third, making lateral suture invisible from above; posterior lateromarginal setae absent; elytra strongly convex, nearly as long as fore body (including mandibles), humeral shoulders roundly angulate, lateral sides smooth; striae reduced but more or less traceable; three dorsal and the pre-apical setae present; marginal umbilicate series not aggregate, only 2^nd^ pore close to marginal gutter; 1^st^ pore of humeral group shifted backward and about level to 5^th^ stria, a little behind 2^nd^; 4^th^ distant from 3^rd^; both pores of middle group lying close to each other; legs moderate for cave trechines, tibiae without longitudinal furrows externally; protarsi in male not modified; ventrite VII bisetose in male, 4-setose in female; male genitalia minute, well-sclerotized, moderately curved in middle part, apex slightly raised and pointed in lateral view; basal part quite large, sagittal aileron present; inner sac armed with a thin and scale-covered copulatory piece; parameres large but short, each bearing three long setae at apex.

#### Remarks.

It is not easy to determine the taxonomic position of this new genus. Several generically important characters of *Tianeotrechus* gen. n. do not exist in the other genera of Chinese Trechini: a quadridentate right mandible, invisible pronotal lateral borders and the presence of three dorsal pores on each elytron. We hope more discoveries in the near future will be able to shed additional light to clarify this problem.

#### Etymology.

Tian’e + Trechus, in reference to the provenance of the type species from Tian’e County. Gender masculine.

#### Range.

China (northern Guangxi) (Fig. 5d).

### Tianeotrechus trisetosus

Taxon classificationAnimaliaColeopteraCarabidae

Tian & Tang sp. n.

http://zoobank.org/CDEDE83E-8CC2-4BAE-A706-45020D2D6265

#### Holotype.

Male, Cave Bahao Dong, Gandong Village, Bala Xiang, Tian’e County, northern Guangxi, 24°55'57.10"N/107°02'40.80"E, 686 m in altitude, VIII-7-2015, Mingruo Tang leg., in SCAU; paratypes: 4 males and 1 female, ibid., in SCAU.

#### Diagnosis.

A medium-sized trechine, with shiny and robust body, moderated appendages, convex pronotum and elytra, and elongated elytra which have round shoulders and reduced striae.

#### Description.

Length: 5.6–5.7 mm (mean 5.66), width: 1.6 mm. Habitus as in Fig. 9.

Body brownish red, with palps, antennae and tarsi pale; frons, vertex and underside of head, pronotum, inner part of elytra glabrous, propleura and prosternum glabrous; genae, lateral parts of elytra, meso- and metasterna, and visible ventrites clothed with short pubescence; legs densely pubescent, covered with longer setae; microsculptural engraved meshes moderately transverse on head, strongly striate on pronotum and elytra.

Head elongate, much longer than wide, HLm/HW = 1.78–1.80 (mean 1.79), HLl/HW = 1.29–1.34 (mean 1.32); frons and vertex moderately convex; frontal furrows fairly long but incomplete, nearly parallel-sided, albeit slightly divergent posteriad, ending near neck constriction; genae barely expanded laterally, both sides held almost parallel; anterior and posterior supra-orbital pores located in the middle of genae and near neck constriction, respectively, distance between anterior and posterior pores much less than that between anterior pores; clypeus 4-setose, labrum transverse, widely but shallowly emarginated at front margin, 6-setose; mandibles distinctly curved at apex; palps thin and moderately long, 3^rd^ and 4^th^ maxillary palpomeres, and 3^rd^ labial palpomere glabrous, 2^nd^ labial palpomere 4-setose; penultimate palpomere evidently longer than apical one of labium, slightly longer in maxilla; suborbital pores on ventral side of genae, located closer to submentum than to base of head; antennae extending to about apical 3/4 of elytra, pubescent from 2^nd^ in apical half; 1^st^ antennomere stout and bearing several setae, slightly shorter than 2^nd^; 3^rd^ 1.6 times longer than 2^nd^; 3^rd^, 4^th^ and 5^th^ subequal in length, then gradually shortened from 6^th^ to 10^th^; 11^th^ as long as 9^th^.

Prothorax expanded due to propleura, but concealed dorsally by pronotum, the latter being more strongly tumid laterally; pronotum longer than wide, PnL/PnW = 1.22-1.32 (mean 1.27); shorter than head with mandibles, PnL/HLm = 0.89-0.94 (mean 0.91); wider than head, PnW/HW = 1.24-1.32 (mean 1.28); base narrower than front, PbW/PfW = 0.885-0.894 (mean 0.889), both nearly straight and unbeaded; widest before middle, lateral margins of pronotum invisible from above; anterior lateromarginal pores present, located at about apical third; middle line fine; frontal impression faint, depressed medially, basal transverse sulcus well-marked; disc strongly convex. Scutellum small and short.

Elytra elongate ovate, much longer than wide, EL/EW = 1.72-1.75 (mean 1.74); as long as head (including mandibles) plus pronotum; much wider than pronotum, EW/PnW = 1.80-1.88 (mean 1.83); widest at about 3/7^ths^ from base; prehumeral part short, humeral angles rounded; lateral sides smooth and well-beaded, ciliated throughout; disc strongly convex except for a small area near base just behind scutellum, the latter being somewhat depressed; striae more or less obliterated but traceable, intervals slightly convex; base not bordered; basal pores on either side of scutellum, close to basal margin; three dorsal pores present on 3^rd^ stria, located at about 1/6^th^, 2/5^ths^, 2/3^rds^ and 5/6^ths^ of elytra from base, respectively; pre-apical pore lying at about 5/6^ths^ of elytra, at site of junction of 2^nd^, 3^rd^ and 4^th^ striae, much closer to elytral suture than to apical margin; humeral group of marginal umbilicate pores not aggregated, 1^st^, 2^nd^ and 3^rd^ pores forming an equilateral triangle, 4^th^ widely distant from other three, 2^nd^ close to, 4^th^ far from, marginal gutter; middle group located behind middle of elytra, close to each other, distant from marginal gutter; apical pore minute, placed near elytral apex.

Legs moderately long, tibiae not longitudinally furrowed, hind tibia as long as elytral width; protarsi short; 1^st^ tarsomere shorter than, or subequal to, or longer than 2^nd^-4^th^ tarsomeres combined in pro-, meso- and metatarsi, respectively; 4^th^ tarsomere wider than long in fore leg, as long as wide in middle leg, and evidently longer than wide in hind leg. Ventrites IV-VII each with one pair of setae.

**Figure 10.**
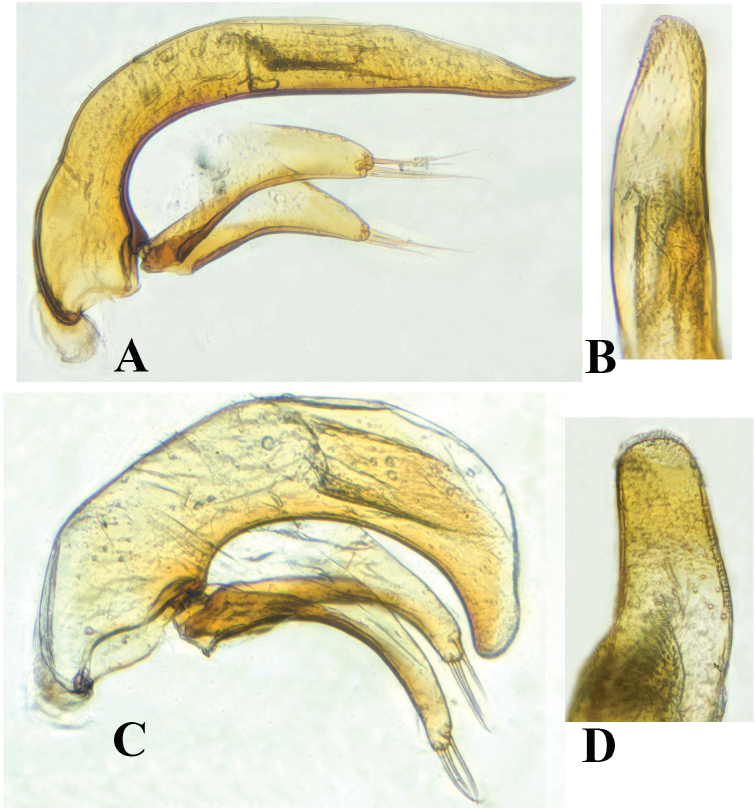
Male genitalia **A** and **C** median lobe, lateral view **B** and **D** apical lobe, dorsal view **A** and **B** *Tianeotrechus trisetosus* Tian & Tang, gen. n., sp. n. **C** and **D** *Wanhuaphaenops zhangi* Tian & Wang, gen. n., sp. n.

Male genitalia (Fig. 10A, B): The median lobe of aedeagus well-sclerotized, small and slender, moderately curved ventrally in middle part, pointed at apex in lateral view; in dorsal view, apical lobe roundly broad at apex, nearly parallel-sided; base widely opening, with a large and thick sagittal aileron; parameres broad, much shorter than median lobe.

#### Etymology.

To refer to the presence of three dorsal pores on elytron.

#### Distribution.

**Figure 11.**
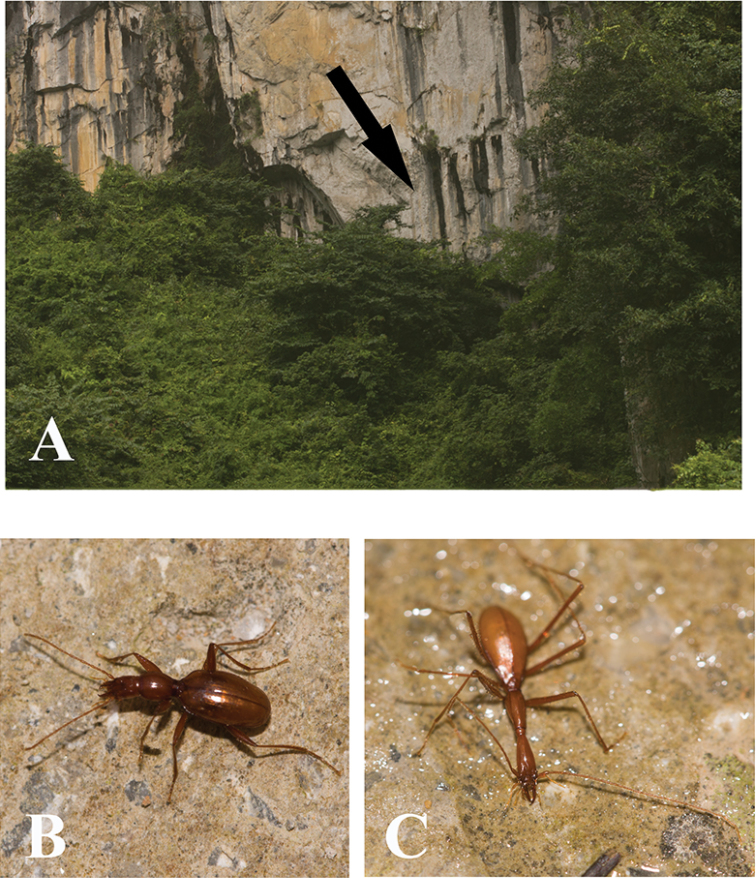
Cave Bahao Dong, type locality of *Tianeotrechus trisetosus* Tian & Tang, gen. n., sp. n. **A** outside cave, arrowhead shows the entrance **B** a wandering individual of *Tianeotrechus trisetosus* in cave **C** a wandering individual of *Dongodytes giraffa* Uéno, 2005, in cave.

China (Guangxi) (Fig. 5d). Known so far from the limestone Cave Bahao Dong, southern Tian’e County (Fig. 11A).

The cave opens below a hill, surrounded by trees and bushes and is invisible from outside. The entrance is large, but the length remains unknown. It is deep and hardly accessible, accumulated by random ripraps; it takes the cavers about an hour to reach the underground river which runs through the deepest part of the cave. All of the type series were collected under stone in twilight and transition zones, thirty to fifty meters from the entrance. It is sympatric with *Dongodytes giraffa* Uéno, 2005 (Fig. 11B, C).

### Huoyanodytes

Taxon classificationAnimaliaColeopteraCarabidae

Tian & Huang gen. n.

http://zoobank.org/1A50BCBA-6A05-4D77-8E78-3EC6C157CAC6

#### Type species.

*Huoyanodytes tujiaphilus* Tian & Huang, sp. n. (Cave Tujiamei Dong, Longshan, Hunan)

#### Diagnosis.

Large-sized, semi-aphaenopsian beetles, with elongated and tube-like head, long fore body, bidentate right mandible, bisetose mentum, well defined labial suture, tubiform and tumid prothorax, five pores in the humeral group of the marginal umbilicate series and disappeared elytral striae.

#### Generic characteristics.

Large-sized, semi-aphaenopsian trechine, eyeless, unpigmented and apterous; fore body longer than elytra; head tube-like, parallel-sided, without neck constriction; much longer than wide, head (including mandible) as long as prothorax; mandible elongate, right mandible bidentate; ligula multisetose; submentum 10-setose, mentum bisetose, each of abdominal ventrites IV-VII 4-setose; labial suture clear, well separating mentum and submentum; frontal furrows short, subparallel-sided, two pairs of supra-orbital pores present; antennae long, extending to a little before elytral apex; prothorax elongate, somewhat tubiform, propleura distinctly tumid and thus visible from above; both fore and hind pronotal angles obtusely rounded; elytra ovate, strongly convex, making marginal side partly concealed and invisible from above; humeral angles rounded, widest at about middle, striae completely missing; apex broadly rounded; two dorsal and the pre-apical pores present on each elytron, humeral group of umbilicate marginal pores composed of five pores, middle group backwardly located, at about apical third of elytra; femora more dilated near subapex; tibiae long and slender, without longitudinal grooves externally.

#### Remarks.

Again, the affinities of *Huoyanodytes* gen. n. are bound to remain obscure. Its tube-like head, the more dilated subapically femora, the very convex pronotum and elytra, and the peculiar elytral chaetotaxy are the apomorphies that make it unrelated to any other genera so far known in China. It must be pointed out that it is the first example in a trechine beetle which humeral group of umbilicate marginal pores as composed of five pores, instead of four.

#### Etymology.

Huoyan+dytes, to refer to this genus occurring in Huoyan Karst. Gender masculine.

#### Range.

China (northwestern Hunan) (Fig. 5e).

### Huoyanodytes tujiaphilus

Taxon classificationAnimaliaColeopteraCarabidae

Tian & Huang sp. n.

http://zoobank.org/C0AA3DF1-1854-4934-988E-B0A89C0E3B7C

#### Holotype.

female, Cave Tujiamei Dong, Huoyan Karst, Huoyan Xiang, Wulongshan Geopark, Longshan County, NW Hunan Province, China, 29°12'20.11"N/109°18'37.21"E, 427 m in altitude, VII-3-2014, leg. Mingyi Tian, Weixin Liu, Haomin Yin, Sunbin Huang & Xinhui Wang, deposited in SCAU.

#### Diagnosis.

A large cavernicolous beetle, with light dark brown fore body, light brown elytra, tubiform head and prothorax, strongly convex elytra and 4-setose on each of visible abdominal ventrites.

#### Description.

**Figure 12.**
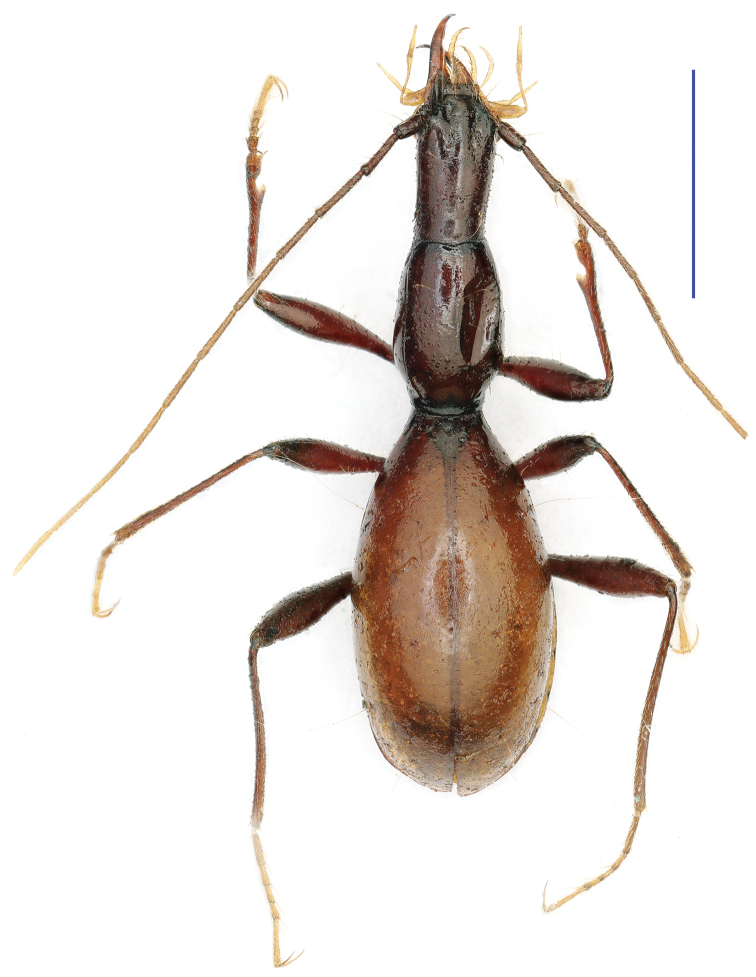
Habitus of *Huoyanodytes tujiaphilus* Tian & Huang, gen. n., sp. n., holotype, female. Scale bar: 2.0 mm.

Length: 7.0 mm including mandibles, width: 2.0 mm. Habitus as in Fig. 12.

Head, pronotum legs excluding tarsi, antennomeres 1-2 light dark brown, elytra, antennomeres 3-11 light brown, palps pale; upper- and underside of head, pro-, meso- and metasterna sparsely covered with rather long setae; elytra glabrous; pronotum with two short hairs in middle portion along mid suture; microsculptural engraved meshes moderately transverse on head, vanishing on pronotum, and strongly transverse on elytra.

Body quite large sized, rather stout, head (including mandibles) plus pronotum slightly longer than elytra, (HLm+PnL)/EL = 1.03.

Head evenly slender, much longer than wide, HLm/HW = 2.90, or HLl/HW = 2.08, genae well-developed and elongated, making head tube-like, nearly parallel-sided; frons, vertex and genae moderately convex; frontal furrows wide and deep, but short, ending at about middle of head from labrum, almost parallel to each other; anterior supra-orbital pores located at about basal 4/7^th^ of head, lateral to frontal furrow and a little before its ending points, posterior ones located at about basal 1/5^th^ of head excluding mandibles; distance between anterior pores as great as that between anterior and posterior pores of each side; clypeus 8-setose; labrum strongly transverse, straight at frontal margin, 6-setose; mandibles long and thin, gently incurved in apical half and distinctly unciform at apex; labial suture clear; mentum widely and deeply concave at base, bisetose, mental tooth simple, blunt at apex; submentum 10-setose; ligula 10-setose, setae being short; palps elongated, slender and subcylindrical, 3^rd^ maxillary palpomere longer than 4^th^, both glabrous; 2^nd^ labial palpomere longer than 3^rd^, bisetose at inner margin, and with two additional setae in subapical and apical parts, respectively; antennae long and pubescent, 1^st^ antennomere stouter, about 2/3^rds^ as long as 2^nd^, which is about 3/4^ths^ as long as 3^rd^, 4^th^ slightly longer than 3^rd^, 5^th^ longest, slightly longer than 4^th^, 6^th^-11^th^ as long as 4^th^; head (including mandibles) plus pronotum slightly longer than elytra.

Prothorax barrel-shaped, longer than wide, PrL/PrW = 1.53, widest at about third from base; longer or shorter than head excluding or including mandibles, PrL/HL = 0.76 or 1.09; much wider than head, PrW/HW = 1.45; propleura distinctly tumid, wholly visible from above; wider than pronotum, PrW/PnW = 1.17; pronotum much longer than wide, PnL/PnW = 1.79, wider than head, PnW/HW = 1.24; subparallel-sided, but narrowly and broadly contracted at both ends, making front and hind angles round off, albeit front ones fairly angulate; lateral margins not beaded; PrW/PnW = 1.17; base nearly as wide as front, frontal margin not beaded, finely emarginated in the middle, basal margin widely beaded and nearly straight; both fore and hind lateromarginal setae placed a little mesal to dorsolateral suture, at about basal fourth and apical fifth of pronotum, respectively; disc slightly convex; median line clear, reaching both ends; both transverse impressions not well-marked. Scutellum small and short.

Elytra ovate-oblong, strongly convex; twice as wide as prothorax, much longer than wide, EL/EW=1.89; widest a little behind middle, lateral margins smooth throughout, neither ciliated nor dentate; without prehumeral angles; apex broadly rounded; striae completely disappeared; two dorsal pores present on the location of 3^rd^ stria, at about basal 2/7^ths^ and 3/5^ths^ of elytra, respectively; pre-apical pores located at about apical 2/11^ths^ of elytra; basal pore present, a little distant from scutellum; humeral group of marginal umbilicate pores not aggregated, composed of five pores, 1^st^ pore transversally removed mesad and backward, at a little behind level to 2^nd^, but a little before the anterior dorsal pore; 3^rd^ pore close to 2^nd^; 3^rd^, 4^th^ and 5^th^ pores widely and equidistantly located; 6^th^ and 7^th^ pores of middle group shifted behind, lying at about apical fourth of elytra; apical group composed of three pores, apical pore located closer to suture than to elytral margin; only 2^nd^ and 9^th^ pores close to marginal gutter, others widely distant from the gutter.

Legs moderately long, femora gradually dilated from base towards subapical portions, then suddenly narrowed towards apices, covered with sparse, long and erect setae; tibiae and tarsi covered with dense and short hairs; tibiae thin, without longitudinal grooves; protarsi short, 1^st^ tarsomere wider than others, longer than 2^nd^and 3^rd^ combined, but shorter than 2^nd^-4^th^ combined; meso- and metatarsi longer, 1^st^ tarsomere as long as 2^nd^-4^th^ combined.

Male: Unknown.

#### Etymology.

tujia + philus, to refer to the fact that the new species is occurring in the country of Tujia people.

#### Distribution.

China (Hunan)(Fig. 5e). Known only from the limestone Cave Tujiamei Dong, Wulongshan Geopark, Longshan County, northwesternmost Hunan Province.

**Figure 13.**
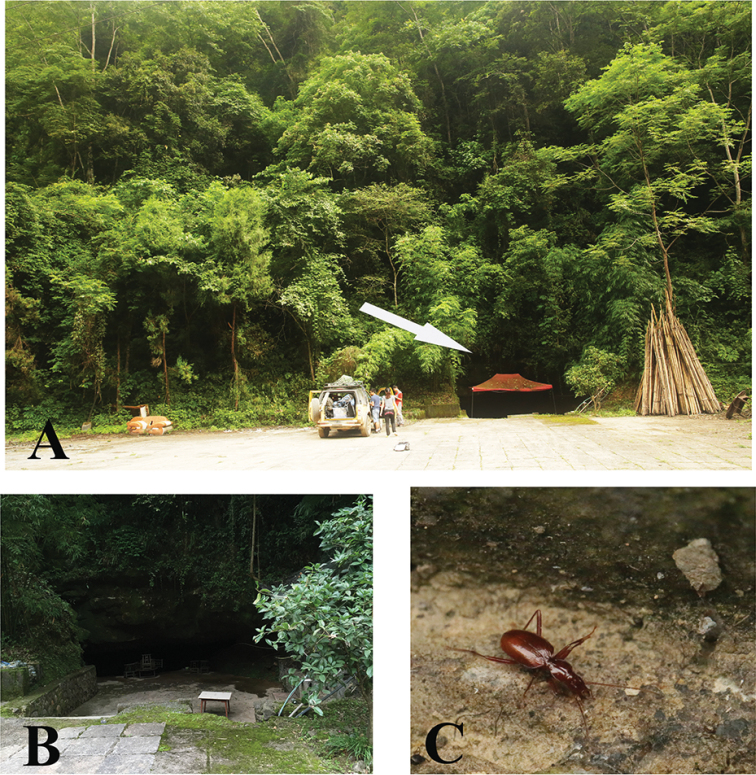
Cave Tujiamei Dong, type locality of *Huoyanodytes tujiaphilus* Tian & Huang, sp. n. **A** environ outside cave, arrowhead showing the site of entrance **B** entrance **C** a wandering individual of *Cathaiaphaenops delprati* Deuve, 1996, a sympatric trechine beetle of *Huoyanodytes tujiaphilus* Tian & Huang, sp. n.

This cave (Fig. 13A, B) lies very close to Feihu Dong, the longest cave in Huoyan Karst, along the main road, and opposite Tujiamei Restaurant. This is a water source cave, with a small underground stream running throughout, the length still being unknown. It is highly moist and muddy. We surveyed as long as about 400 m in the cave, and collected the unique specimen in the dark zone when it was wandering on the wall. The other three trechine species found in the cave are *Cathaiaphaenops delprati* Deuve, 1996 (Fig. 13C), *Sinotroglodytes bedosae* Deuve, 1996, and *Toshiaphaenops ovicollis* Uéno, 1999. We visited this and adjacent caves in July, 2015 in order to find more specimens of this interesting beetle, but failed to catch anything.

### Wanhuaphaenops

Taxon classificationAnimaliaColeopteraCarabidae

Tian & Wang gen. n.

http://zoobank.org/C04A6404-CAD3-421C-97C8-5848046875BB

#### Type species.

*Wanhuaphaenops zhangi* Tian & Wang, sp. n. (Cave Songjia Dong, Chenzhou, Hunan).

#### Diagnosis.

Medium-sized, aphaenopsian beetles, body elongate, with short antennae and quite long legs, slender head, reduced frontal furrows, bisetose mentum, clear labial suture, short and tumid prothorax, elongated elytra and bisetose on each of abdominal ventrites.

#### Generic characteristics.

Medium-sized, aphaenopsian type trechine, eyeless, unpigmented and apterous; body very strongly elongate, highly modified morphologically, albeit antennae rather short; head typically aphaenopsoid, extremely elongated as in *Dongodytes* Deuve, 1993 or some members of *Sinaphaenops* Uéno & Wang, 1991, much longer than wide, with short and incomplete frontal furrows ending at about middle of head from clypeus, two pairs of supra-orbital pores present, both anterior and posterior pores widely spaced; mandibles moderately long, well-developed, right mandible tridentate; labial suture clear; mentum bisetose, distinctly concave, tooth moderately long, thick and blunt at apex; submentum provided with a row of seven (or eight in a male individual) setae, median one much shorter than others; antennae quite short, extending to about middle of elytra; prothorax distinctly shorter than head, longer than wide, propleura strongly tumid, visible from above; pronotum subquadrate, base nearly as wide as front, both anterior and posterior lateromarginal setae present; elytra strongly elongate, slightly longer than head (including mandibles) plus prothorax; widest behind middle, marginal sides smooth throughout, but ciliate in humeral angle area; humera distinctly angulate; disc moderately convex, rather flat near base, striae well-defined or obliterated, two dorsal and the pre-apical pore present; humeral pores of marginal umbilicate series not aggregated, middle group not close to each other; legs fairly long, 1^st^ protarsomere in male modified, with a tiny apical denticle inward; tibiae without longitudinal furrow externally; ventrite VII with two pairs of setae in both sexes; aedeagus minute, well-sclerotized, short and broad, strongly arcuate, apex blunt, basal part large, with a small sagittal aileron, inner sac with a fairly large copulatory piece, parameres long, right one longer than left one, broad at apex, each bearing three long apical setae.

#### Remarks.

The true affinities of *Wanhuaphaenops* gen. n. likewise remain uncertain. Probably the closest match is *Shenaphaenops* Uéno, 1999 (from northwestern Guizhou Province) because both share several important characters: a wholly pubescent body, humera strongly angulate, right mandibles tridentate, only 1^st^ protarsomere modified in male, two pairs of supra-orbital pores present on head, two dorsal and the pre-apical pores present on elytron, and ventrite VII 4-setose. However, *Wanhuaphaenops* gen. n. is easily distinguished from *Shenaphaenops* by the following characters: (1) head much more elongated, with anterior supra-orbital pore widely distant from posterior one, and labial suture clear (reverse in *Shenaphaenops*); (2) antennae much shorter than in *Shenaphaenops*, in which these extending to nearly elytral apex; (3) pronotal posterior lateromarginal setae present in *Wanhuaphaenops* gen. n., but absent in *Shenaphaenops*; (4) aedeagus stouter and strongly arcuate in *Wanhuaphaenops* gen. n., with each paramere bearing three apical setae(Fig. 10C, D), versus aedeagus being slender and slightly arcuate, with each paramere bearing two apical setae in *Shenaphaenops* (Fig. 4C, D).

*Wanhuaphaenops* gen. n. might also be found related to the genus *Sinaphaenops* Uéno & Wang, 1991, one of the most highly modified genera among the Chinese cave-dwelling trechines which ranges from west, southern Guizhou and northernmost Guangxi. Both share a somewhat similar body configuration, but *Wanhuaphaenops* gen. n. is much smaller and less troglomorphic than *Sinaphaenops*, the appendages being much shorter, and only one joint of protarsi (1^st^ protarsomere) is modified in the male, versus two, and a different elytral chaetotaxy.

#### Etymology.

As Cave Songjia Dong represents one branch of the Wanhuayan cave system, the name of this new genus refers to the occurrence of this aphaenopsian beetle in Wanhuayan caves. Gender masculine.

#### Range.

China (southern Hunan) (Fig. 5f).

### Wanhuaphaenops zhangi

Taxon classificationAnimaliaColeopteraCarabidae

Tian & Wang sp. n.

http://zoobank.org/C164C788-0F9A-439E-A31A-B774DC7BB2DD

#### Holotype.

male, Cave Songjia Dong, Beihu Qu, Chenzhou, southern Hunan Province, 25°40'08.05"N/112°53'59"E, 493 m in altitude, VIII-25-2015, Xinhui Wang, Sunbin Huang, Mingruo Tang & Pingjing Yang leg., in SCAU; paratypes: 9 females & 9 males, ibid., in SCAU.

#### Diagnosis.

A slender and brown cave beetle, with a collar-like neck constriction on head, fairly long fore body which is slightly shorter than elytra, long head which is distinctly longer than prothorax, and distinct humeral angles of elytra.

#### Description.

**Figure 14.**
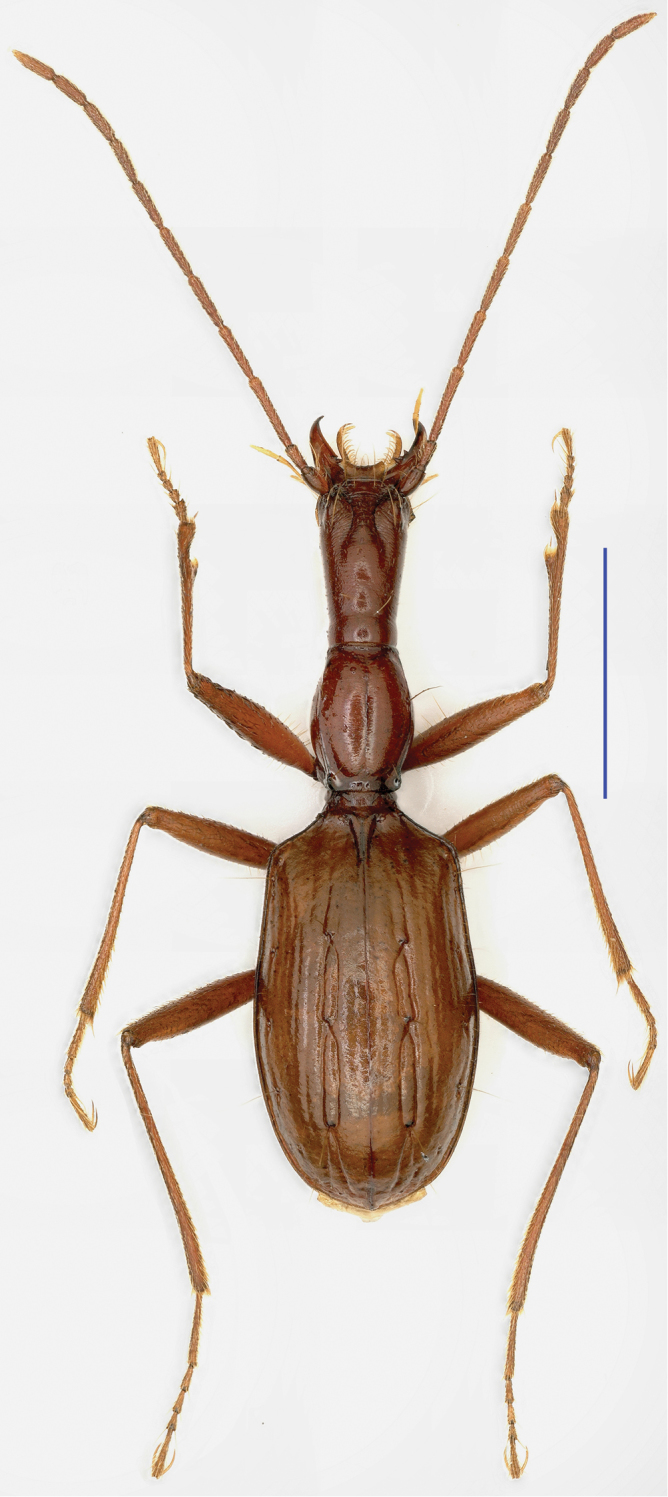
Habitus of *Wanhuaphaenops zhangi* Tian & Wang, gen. n., sp. n., holotype, male. Scale bar: 2.0 mm.

Length: 5.4–5.8 mm (mean 5.6); width: 1.4–1.6 mm (mean 1.5). Habitus as in Fig. 14.

Body wholly brown, upper surface covered with sparse and minute pubescence, genae and underside of head with some longer setae, abdominal ventrites covered with denser minute pubescence, prosternum, propleura and meso- and metasterna glabrous; legs densely pubescent; microsculpture composed of finely, densely and strongly transverse meshes on upper and underside surfaces. Body elongated, fore body, including mandibles slightly shorter than elytra.

Head strongly elongated, HLm/HW = 2.37–2.5 (mean 2.44), HLl/HW = 1.89–1.94 (mean 1.91), widest at about third of head from labrum, then gently narrowed towards a collar-like constriction of the neck; anterior supra-orbital pore level to the widest point, posterior one at about 1/5^th^ of head from base, strongly behind end of frontal furrows; distance between anterior and posterior pores greater than that between both anterior pores; frontal furrows fine but well-defined, short, nearly parallel-sided in the middle, divergent posteriad, but then convergent before ending points; anterior supra-orbital pores located at the level of mid frontal furrows, posterior ones near collar-like constriction, distance between both posterior pores about half as that between anterior and posterior pores of either side; frons and vertex moderately convex; clypeus quadrate, 4-setose; labrum transverse, widely but shallowly emarginated at front margin; mandibles gently unciform at apex; palps fairly slender, 3^rd^ and 4^th^ maxillary palps glabrous, subequal in length; 2^nd^ labial palp distinctly longer than 3^rd^, with two setae at inner margin, and 2–3 additional ones in subapical part, 3^rd^ glabrous; suborbital pores on ventral side, near a collar-shaped beaded neck; 1^st^ antennomere thick, as long as 2^nd^; 3^rd^ antennomere longest, 2.5 times as long as 1^st^; 4^th^–7^th^ and 11^th^ slightly longer than 8^th^–10^th^.

Prothorax shorter than head, PrL/HLm = 0.60–0.67 (mean 0.63), PrL/HLl = 0.75–0.86 (mean 0.80); but much wider, PrW/HW = 1.11–1.17 (mean 1.14), longer than wide, PrL/PrW = 1.29–1.43 (mean 1.34), widest at about 3/7^ths^ from base; pronotum much longer than wide, PnL/PnW = 1.35–1.58 (mean 1.45), slightly wider than head, PnW/HW = 1.05–1.06 (mean 1.05); widest behind middle, sides beaded, gently narrowed both distad and basad, distinctly sinuate before hind angles, both front and hind angles obtuse, albeit hind ones more angulate and distinctly reflexed; anterior lateromarginal setae at about apical 2/5^ths^, posterior ones close but a little before hind angles, distinctly shorter than the formers; base slightly wider than front, PbW/PfW = 1.05–1.07 (mean 1.06), both nearly straight, front thickly and widely bordered, base unbordered; disc convex; middle line deep, connected to both front and basal impressions. Scutellum short and small.

Elytra fairly long and elongate ovate, much longer than wide, EL/EW = 1.78–1.82 (mean 1.80), slightly longer than fore body; widest at about 4/9^th^ from apex, lateral margins finely beaded from base to finish just before apex, finely ciliate throughout, but remarkably distinct in angular area, nearly straight before and behind humeral angles; base not bordered; disc convex, but basal or humeral area distinctly depressed and almost flat; 2^nd^ and 3^rd^ striae well-marked and complete, others more or less obliterated; all dorsal and pre-apical setiferous pores located exactly on interrupted and junction points of 2^nd^ and 3^rd^ striae, making 3^rd^ interval with three regular longitudinal meshes between the pores; basal pores located near base, along both sides of scutellum; anterior and posterior dorsal pores at about basal third and middle of elytra, respectively, pre-apical pore at apical fourth of elytra, much closer to suture than to apex of elytra; humeral set of marginal umbilicate pores not aggregated, 1^st^-3^rd^ pores equidistantly located, quite near the marginal gutter, 4^th^ distant from 3^rd^; 5^th^ and 6^th^ isolated from each other, though 5^th^ closer 6^th^ than to 4^th^.

Legs thin and fairly long, femora moderate, tibiae not longitudinally furrowed, hind tibia slightly longer than elytral wide; protarsi short; 1^st^ tarsomere shorter than, or subequal to, or longer than 2^nd^-4^th^ tarsomeres combined in pro-, meso- and metatarsi, respectively.

Male genitalia (Fig. 10C, D): The median lobe of aedeagus very small, but well-sclerotized, with a small but distinct sagittal aileron and a fairly large copulatory piece; parameres well-developed.

#### Etymology.

This species is named in honour of Prof. Yuanhai Zhang (Institute of Karst Geology, Chinese Academy of Geological Sciences, Guilin), who was leading the cave exploration project at Wanhuayan in late August 2015, one of the results being the discovery of this interesting species.

#### Distribution.

China (Hunan) (Fig. 5f). Known only from the limestone Cave Songjia Dong, in the Wanhuayan cave system.

**Figure 15.**
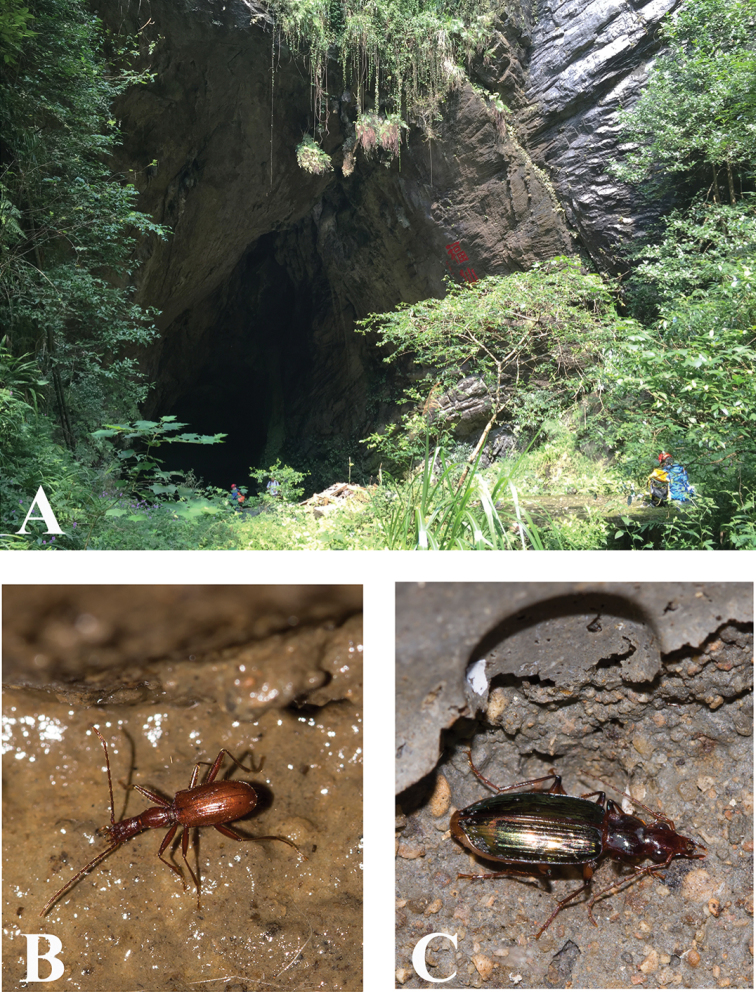
Cave Songjia Dong, type locality of *Wanhuaphaenops zhangi* Tian & Wang, gen. n., sp. n. **A** entrance **B** a wandering individual of *Wanhuaphaenops zhangi* **C** a platynine *Colpodes* beetle in the cave.

Songjia Dong is the upper part of the Wanhuayan cave system, about 10 km away from the main entrance of the Cave Wanhuayan. It is a water cave, with the entrance being as big as that in Wanhuayan (Fig. 15A). The beetles were collected in a dark area about 80 m deep from the entrance (Fig. 15B). A *Colpodes* species, a trogloxene, was also found in this cave (Fig. 15C).

## Supplementary Material

XML Treatment for Shiqianaphaenops

XML Treatment for Dianotrechus

XML Treatment for Dianotrechus gueorguievi

XML Treatment for Tianeotrechus

XML Treatment for Tianeotrechus trisetosus

XML Treatment for Huoyanodytes

XML Treatment for Huoyanodytes tujiaphilus

XML Treatment for Wanhuaphaenops

XML Treatment for Wanhuaphaenops zhangi
